# Acknowledgement to reviewers

**DOI:** 10.1530/JME-76-A1

**Published:** 2026-01-19

The editorial board wishes to thank the following individuals who have helped to review manuscripts for *Journal of Molecular Endocrinology* during 2025. Their assistance is gratefully acknowledged.

**Table:** 

K Aagaard
J F Abbade
R Abdala
M M Abd-Alhaseeb
B Abderrahman
G A Abruzzese
A Adaikalakoteswari
S Adam
R Adelaiye-Ogala
E O Adewuyi
S A Adham
D Africander
R Agoro
C Aguayo-Mazzucato
B-C Ahn
P Aich
F Alemi
R Alen-Alonso
R H Ali
H Ali Nagi Al-Jamal
K Aljerian
J Almaça
M I Almeida
M R Alvares-da-Silva
M G Alves
A Amaro
J P d A Amorim
J H An
Y An
J H Andersen
G Andican
C L Andoniadou
P Andrikopoulos
J Arab
N Arata
H Arefanian
C Arehart
A Armani
I Artner
H H Atteia
M Auer
J Auwerx
S Azhar
K Azukaitis

Q Ba
K Bacos
R Baker
T M Ballhause
J P Banga
L Barazzuol
J Barclay
C Barosa
J Barrera-Chimal
A A Bartlett
M Bartoli
S Bath
C Beall
W Beck
D Becú-Villalobos
A Belgorosky
D Bell
M A Belury
S Benfeito
R G Bennett
A Benrick
A Ben-Shlomo
C Berasain
D J Bernard
C Bernardini
D E Berryman
M Bertagnolli
A Berthier
F-R Bertin
P Bertolino
V M Betz
R Beydoun
N P Bgatova
R Bhatnager
M Bhattacharya
X Bi
E Bindi
C Bishop
C S Blesson
F F Bloise
S Blouin
P A Boer
W B Bollag
S Borghi
D Boriboonhirunsarn
N Borradaile
P Borrow
C F Bortolatto
J Boucher
L Bourebaba
K E Boyle
J Breves
B Brew
S Briuglia
S Brooks
K Brown
T Brue
E Bruinstroop
G Bruno
N Buisine
G Buitrago
S Bulun
K Bunte
G W Burns
T Burrinha
K T Butterworth
A Byrd

G Calin
L Calleros
I Camacho-Arroyo
J Campbell
M Campbell
J P Camporez
M Candrlic
E Cano-Europa
O Cantoni
S Cao
B Caramelo
D Cardinali
A Care
B Caroccia
R Carr
A Carré
R Carroll
E Casado
R C V Casarin
L Casarini
J P Castaño
E Cavalier
V M Ceglarek
K Cesoraletti
T Challa
P Chaloulos-Iakovidis
L F Chan
M Chan
T-C Chang
P Chanson
M Charlton
S C Chattipakorn
N Chattopadhyay
C Chatzakis
U Chaudhari
S Chaugule
C-M Chen
H Chen
J Chen
L Chen
L-L Chen
P Chen
S Chen
X Chen
Y Chen
Y Q Chen
Z-J Chen
S-y Cheng
O V Chernikov
V Chesnokova
E Chin
A M Chindris
V Chiofalo
W-C Chiu
T Chivese
C Chlebek
C-I Choi
J W Choi
J-Y Choi
A Chougule
G Christopher
C T Chu
S Chung
A A A Chuturgoon
E Cianflone
J Claria
C M Clay
M S Clerget-Froidevaux
K Cockerham
J Cohen-Tannoudji
S Conde
V Condello
B Conway
M Correia
T Coskun
A Cowart
A J Cowin
P Crepaldi
C Cruz
J S M Cuffe
Y Cui
B Culbert
T Cunha-Oliveira
R Cunningham
S Cusack

G da Silva Xavier
E M Dalmarco
T F Davies
B De La Torre
A F de Melo
R de Molon
E G de Moura
L P de Moura
F Deepika
R Deka
M Dekker Nitert
C Dela Cruz
N Delk
S Delorme
T M Dencic
H Deng
J Deng
Y Deng
Z Deng
F Deschamps
I W Deveson
W S Dhillo
S Dhindsa
L Di Croce
S Dias
R Diaz-Molina
A M Diehl
W Dik
G K Dimitriadis
K Dingle
W L Dissanayaka
K Dobrzyń
C Dogaru
G Dolton
X C Dong
S Dooley
R Douglas
R S Douglas
A Drakesmith
K M A Dreijerink
M V Dubinin
Q R Ducarmon
R Duggavathi
C Duță

R Eastell
M K Ediriweera
M Eftekhar
E K Egger
I El Yazidi-Belkoura
R Elisei
W H El-Maadawy
S M Eraky
O Escribano
A Estienne

B Fam
L Fang
M Fang
S Fang
J N Farr
C Faul
A T Fazleabas
C Feng
G Fenna van Breda
A Feraco
F L Fernandes-Rosa
P Fernandez-Garcia
C Fernandez-Hernando
S M Ferrari
C D Figueroa
J-B Fini
Z Fisar
C E Fletcher
T Fløyel
G L Forni
M Foti
L Fozzatti
E Francis
G Franzoni
G Fruhbeck
L Fugazzola
R Fujii
S Fujisaka
M Fukami
S Fukumoto
T Fukumoto
S Fulzele

S A Gabr
M Galdiero
M R Galdiero
L Gao
M Gao
V Garla
D Garrido
L Gasberg
E L Gastal
G Gembillo
B Geng
P Geraldes
M Gericke
S Gharanei
M Gillingham
L Giovanella
C Godinot
L Goedeke
M Golczak
W Goldner
P Gong
S-q Gong
M O Goodarzi
B Goodpaster
J Goodwin
R Görges
A R Gortázar
C M Gorvin
A Goyal
G Grandl
D Graves
G Grigelioniene
A Gross
A Grotzinger
J Gu
G Guan
R Guan
X Guan
J F Guerreiro
B Guigas
C Guillen
J Guo
S Guo
Y Guo
C G Gutiérrez

J Hadoux
E Hamilton-Williams
G D Hammer
K N Hascup
S M Hashemi
Y Hasija
G M Hatch
E Hattingen
C P Hawkes
M Hazra
A He
H He
Y He
J Heeren
D Heery
A C Heijboer
J T Heiker
Y Hekmatshoar
H Heuer
A Hidalgo
C E Hills
C Hilton
M Hlavackova
B Holder
J M Hollander
L S Holliday
M Holmberg
J J Holst
N Z M Homer
L Hong
T Hong
A R Horvath
N Horwood
Z Hou
N Houreld
V Hoving
L Hoyles
P-S Hsieh
B-G Hsu
J Hu
Y Hu
T Huan
B-M Huang
M Huang
S Huang
Y Huang
Y-T Huang
Z Huang
M O Huising
M L Hull
K Hummitzsch
R Hunt

T Imamura
O Inanami
G Ioannou
W R W Ishak
K Ishino
H Ismael
Y Iwasaki
N Iwata

I Z Jaffe
R Jalbout
J S Jarmasz
J Jaume
V Jendrossek
F-h Ji
J Jiang
X Jiang
Y Jiang
J Jin
Y Jin
R Jockers
Y Johmura
J Jones
S S Jonker
P A Jose
I Jozic

P Kachroo
U B Kaiser
H Kaji
J Kallijarvi
A Kalsbeek
T Kaminski
S Kaneko
A Kannt
H-Y Kao
P Kar
E Karaman
H Karatas
A Kasonga
I A Katayama Rangel
H Katoh
G Kaur
R Kaur
M Kawaminami
B Kazemi
T Kazemi
A Kazlauska
B G Keevil
E T Keller
T Kessler
G L Khatik
S Khosla
S J J Khundmiri
M Kiezun
N Kikyo
J H Kim
S Kim
M Kimura
R D Kineman
H Kirchner
M C Kiremit
J Kirkland
J Klammt
L Knani
K Knapczyk-Stwora
J M Ko
K Kobaly
G Kokeny
B Kong
L Kong
Y Kong
S Kopprasch
S Kousteni
M Kowalewski
A Krag
G E Krassas
K Krause
C C Krieger
K Kubatzky
J C Kuchler
V Kuek
T Kumai
D Kumar
T Kuo
W Kuri-Harcuch

N Lachmann
L Laffin
B Laffitte
L Lahti
M Laloraya
A Lama
L Landsman
C Lange
M Lappas
G Lash
P Lauffer
A C Lay
C Lazzaretti
H-J Lee
J Lee
J H Lee
K Lee
S J Lee
S-Y Lee
Y-H Lee
T Lefebvre
Y Lei
E C R Leonel
L A Lesniewski
B Li
H Li
J Li
M Li
M-D Li
Q-W Li
X Li
Y Li
Z Li
S H Lim
W Lim
I Lima
S M Limbu
H Lin
H-Y Lin
K Lin
L Lin
K E Lines
A Linglart
L Litvinova
A Liu
D Liu
G Liu
H Liu
M Liu
Q Liu
R Liu
Y Liu
Z Liu
J Loffing
K Loh
G Lombardi
F Long
G Lopaschuk
J J Lopez
C Lopez-Camarillo
A Lopez-Valverde
K L Loveland
C Lu
D-Y Lu
H Lu
K-H Lu
M Lu
L Lu
J Luk
Y Luo
C Luongo

L Ma
X Ma
Z Ma
Z F Ma
T MacDonald
S Mader
L Magnoni
P Maiti
A N Malik
X Man
C Manani
C Mando
K C Mansky
A Mansour
W Mao
J P Mardegan Issa
N Marek-Trzonkowska
C Mariash
D E Maridas
L Marinelli
E Marino
M Marinò
G Marone
P-J Martens
A Martinez-Sanchez
F Martins
P Marzullo
P Matafome
S Matos
J Maurer
S Mayerl
J Mayneris Perxachs
S Mazumdar
S McArthur
C J McCabe
C McCarthy
M McDonald
M E McGee-Lawrence
A M McKillop
Z Mei
Q Meng
L Menichetti
E Menkhorst
M Merchant
Z Michailidou
B S Miller
A Milluzzo
H M Minderhout
S Mirabilii
S M Miraglia
M Mirandola
T Mitic
J Mittag
A Mizokami
L C Moeller
S Mohammadi-Yeganeh
Z Mohammed-Ali
T Moholdt
L Möller
S H Møller
A Molvarec
M K Montgomery
M M Montt Guevara
Y J Moon
M B Moravek
P J Morgan
S Morimoto
B Morte
K G Mountjoy
A J Mouton
J Movassat
J W Mueller
S Mueller
D Mukherjee
S Mukherjee
A C Muller Kobold
M Munshi
N Muratoglu Sahin
K Murotomi
C Muscurel
K Musilek
L Myatt

L Nadeem
F Naef
R Nafe
Y Nakamura
Y Nan
J Nedergaard
G Nelli
E R Nelson
E Nemeth
A-E Nenciu
L Nicolás-Toledo
X Nie
S E N Nissen
A K Nogueira de Alencar
B J Nolan
S Nölting
T V Novoselova
P Nunes

S O’Mahony
E Oancea
S Ohya
P J Oliveira
A Olmos-Ortiz
G Omuse
L Ongaro
J Oracz
M L Oróstica
Y Otsuka
H Ozawa
L Ozcan
N Ozer

V Padmanabhan
R d P Padovani
A Pal
G Palma
T Palmer
G Panahi
K Pantopoulos
A Pappa
O Pardo
J-H Park
A Parker
H J Parker
R Parsanathan
L Pasquali
M Patankar
R Patarrao
D Pathak
G Pathare
V Patial
S Patterson
N Pavlos
S H S Pearce
J Pearson
U Pecks
F Peixoto
R Peiyao
M K Pekcan
S Pekkala
C G Pellizas
T Peng
Z Peng
J C Peracoli
A Pereda
S Pereira
A V Perkins
W Perng
C Perri
D Peruzzo
M Petersen
A R Pettit
C Pheiffer
A Piekiełko-Witkowska
R Piet
T Piltonen
P Pinge-Filho
Z Piperigkou
M Pisarska
M Pistis
K G Pohler
M Pokorna
K G Poppe
K G Pringle
D Purrahman
E Puxeddu

J Qi
X Qi
Z-M Qian
X Qiao
L Qin
Y Qiu
L Qu
N Quarto

D Rachon
P Rada
R Raghupathy
A Rak
P Ramakrishnan
R Ramanathan
K Ramani
S Ramirez
E Ramirez-Hernandez
K Ramkumar
V Raverot
M L Read
M Rebollo-Hernanz
S Rebouissou
R S Rector
D Regazzo
J Rege
M Reincke
A L A Reis
S Ren
P C N Rensen
L V Rhodes
R Ribeiro
M Riera
Y Rijke
R Ringseis
M M Rinschen
N Rivero-Segura
V Rochira
M Romao-Veiga
C Romero
X Rongfang
P Rorsman
J C Rosa-Neto
S Rose
D Rosendo-Silva
R Rosenfeld
M M Rosenkilde
K Ross
M Rossmann
S Roth
P Roy
H-B Ruan
P T Ruane
I Rubio-Aliaga
P Ruchaya
H Ruohola-Baker

P Sabeti
N Salleh
M Salvi
M Sampaolesi
V Sangkhae
W D Santos
V Sardao
A Sarma
K Sato
D Savic
S Schaefer-Somi
M Schosserer
S J Schrodi
R Schulz
A Schutt
M Schwarzer
W Scott
E S Seber
E Seebach
V Selvaraj
R Semba
R K Semple
A Sewell
J Sfeir
O Shafi
K Shankar
L Shao
M Shao
D Sharon
A Shen
F-M Shen
W-J Shen
V D Sherk
S Shetty
Y Shi
A Shikanov
A O Shpakov
G Shulman
L Sieminska
F d F Silva
R F Simoes
T Simoncini
R A Sinha
B S Siqueira
S Sivaramakrishnan
J T Smith
N Smith
T J Smith
U Smith
D Sobrido-Cameán
S Softic
S Soldano
M J Soler
B-f Song
D Song
J Song
K Song
M Song
Y Song
K Spalding
T Speckmann
J Spetz
K L Spiller
M Srivastava
B Staels
I Stafeev
G Stamatiades
J Stefulj
B Stiles
I Stoian
X L Strudwick
S Sukumari-Ramesh
N Sun
W Sun
Z Sun
L-Y Sung
M Suppli
F Suriano
D Swinkels
A Szeliga

O Tabatabaei-Malazy
T Takahashi
H Takeuchi
R Talbi
P Tammaro
K Tanabe
C-H Tang
D Tang
H Tang
J Tang
H Tange
Y-X Tao
A I Tarasov
E B Tari
A Taylor
L Teimoori-Toolabi
J Teixeira
A Terasmaa
L I Terrazas
D Terris
A Thakur
R Thapa
G Theocharidis
K Thirumurugan
D Thomas
P Thompson
W E Thompson
K L Thornburg
M Thuzar
D Tian
S Tian
X-s Tian
J Tickner
H W Tiemeier
L Tiret
D Tomaszewska-Zaremba
W Tong
D Torella
A G Torres
H Tostivin
H Tostivint
M Trauner
F Tremollieres
E Triplett
M Tripodi
V Trudeau
Y-H Tseng
S Turan
H Turk
A Turnell
M C Turner
S Turolo
J Turrentine

K Uoshima
M Upadhya

T Vallim
B C J van der Eerden
A E van Herwaarden
J J Vandana
G Varricchi
T Vatanen
D Vatner
A I Vazquez
E R Vazquez-Martinez
V Vecchio
E Vegter
R Velmurugan
W A Verri Jr
J F Vettorazzi
G Viana
M H Vickers
A Vidal-Puig
R Videira
I Viegas
M M Vijayan
E Villalobos
L Vitiello
H Vogel

S M Wajner
H Wan
B Wang
D Wang
G Wang
H Wang
J Wang
J-H Wang
K Wang
L Wang
P Wang
Q Wang
S Wang
Y Wang
L S Ward
A J Watkins
M J Watt
S Watts
D J Waxman
M Weber
R Wei
G Weiss
J M Weitzel
M Weivoda
L Wen
S L White
S H White-Springer
J M Wilson
I S Winer
J M Wit
P Witt-Enderby
E Wolf
C K Wong
S K Wong
J Wood
T M Woodruff
D Wootten
R E Wootton
S Wray
A Wu
N Wu
P-H Wu
S-P Wu
Y-L Wu

X Xia
F Xie
L Xie
Y Xin
F-j Xiong
R Xiong
C Xu
G Xu
L Xu
M Xu
Q Xu
S Xu

R K R Yaatvelli
A K Yadav
H Yamada
S Yan
Z Yan
B Yang
F Yang
G Yang
H-C Yang
M Yang
P Yang
S Yang
S-F Yang
X Yang
Y Yang
F Yao
J Yao
Z Yao
A Yaromina
H Yasui
J Ye
N Yi
S A Yi
X Yi
H Yokoi
N Yokoi
S Yonekura
B Yu
J Yu
W Yu
X-J Yu
Y Yu
S Yuan
J W Yun

M Zaidi
J D Zajac
E Zavala
T Zeng
M-C Zennaro
K Zhai
S Zhan
B Zhang
C Zhang
D-w Zhang
H Zhang
J Zhang
M Zhang
Q Zhang
S Zhang
Y Zhang
Z Zhang
G-J Zhao
H Zhao
W Zhao
D Zheng
G Zheng
H Zheng
L Zheng
H Zhou
X Zhou
Z Zhou
Q Zhu
S Zhu
X Zhu
X-X Zhu
S Zraika
D Zyzelewicz

